# Community perceptions on challenges and solutions to implement an *Aedes aegypti* control project in Ponce, Puerto Rico (USA)

**DOI:** 10.1371/journal.pone.0284430

**Published:** 2023-04-17

**Authors:** Carmen L. Pérez-Guerra, Coral Rosado-Santiago, Sue A. Ramos, Karla M. Marrero, Gladys González-Zeno, Julieanne Miranda-Bermúdez, Marianyoly Ortíz-Ortíz, Vanessa Rivera-Amill, Stephen Waterman, Gabriela Paz-Bailey, Liliana Sánchez-González

**Affiliations:** 1 Division of Vector Borne Diseases, Centers for Disease Control and Prevention, Dengue Branch, San Juan, Puerto Rico, United States of America; 2 Ponce Research Institute, Ponce Health Sciences University, Ponce, Puerto Rico, United States of America; 3 Puerto Rico Science, Technology and Research Trust, Puerto Rico Vector Control Unit, San Juan, Puerto Rico, United States of America; Federal University of Agriculture, Abeokuta, NIGERIA

## Abstract

This study characterizes community perceptions on a large-scale project seeking to reduce the population of *Aedes aegypti* mosquitoes and prevent arboviral disease transmission in Ponce, Puerto Rico; and to leverage on these perceptions to make modifications to ensure effective project implementation. In 2017–2018 the team conducted informal interviews, focus groups, and in-depth interviews with leaders and residents of the communities, focusing on challenges and potential solutions to the project implementation. Possible challenges to the project implementation included the lack of geographic consistency between clusters defined by researchers and the participants’ description of the communities’ geographic boundaries. Few children living in the communities could affect the ability of the project to adequately measure arboviral disease incidence. Also, population attrition due to out-migration, and lack of community leaders and communication channels after Hurricane Maria could affect participation in project activities. Lack of trust on strangers was an important challenge due to criminal activity involving violence and drug use in some community areas. Solutions to the identified challenges included identifying emerging leaders and implementing community meetings to promote project activities. The information that community members provided helped us to understand the natural disasters’ impact on population attrition in these communities with a disproportionate impact in younger groups, resulting in an aging population. We identified lack of community organization and leadership and increasing number of abandoned houses that could turn into *Aedes aegypti* breeding sites. The formative work helped to better define the geographic areas that the study would cover, evaluate the acceptability of innovative vector control methods, and identify communication methods used by residents. With this information, challenges and potential solutions in recruiting participants were anticipated, and the community engagement and communications plans were developed. We recommend selecting clusters before research, because opinions towards mosquito control technologies could vary in added clusters.

## Introduction

*Aedes* mosquitoes transmit dengue, Zika, and chikungunya viruses. These have caused major epidemics in tropical areas of the world, representing a great economic burden on public health and healthcare systems [1–7]. In Puerto Rico, dengue has been endemic for decades, while chikungunya and Zika were introduced in 2014 and 2016 respectively, resulting in major outbreaks [8–10]. Traditional control methods for *Aedes aegypti* (*Ae*. *aegypti*) and arboviral infections conducted by public health and municipal authorities in Puerto Rico include street-level spraying of insecticides against flying mosquitoes, collecting debris and used tires that serve as mosquito breeding sites, and educational campaigns directed at the elimination or management of household water containers and mosquito bite prevention. These efforts to reduce mosquito populations have not prevented epidemic virus transmission because, among other reasons, the approaches are not consistently carried out by the public or the government [11–14]. Additionally, street-level spraying and indoor insecticide spraying have contributed to widespread insecticide resistance in *Ae*. *aegypti* mosquitoes [15, 16] in Puerto Rico [17].

Other control methods for *Ae*. *aegypti* were implemented during the chikungunya and Zika epidemics. Projects using autocidal gravid ovitraps (AGO) in the southern and central areas of the island demonstrated reduction in mosquito populations and transmission of chikungunya virus [18–20]. However, control of *Ae*. *aegypti* and the arboviruses at a larger scale have proved challenging in Puerto Rico and worldwide. Thus, the need to evaluate innovative controls for *Ae*. *aegypti* continues. To better implement innovative mosquito control tools, it is necessary to consult communities about their acceptability and use [21–28].

Literature on implementing vector control projects with community participation underscores the benefit of researching the sociocultural environment and organization of communities, their understanding of arboviral disease prevention, and perception on mosquito control strategies [29–38]. For example, public opposition and resistance that halted aerial spraying with Naled insecticide during the 2016–2017 Zika outbreak in Puerto Rico due to misconceptions and mistrust in the process might have been prevented by timely education before aerial spraying began [39–41]. A content analysis of Puerto Rican newspapers showed that “understanding public perceptions, providing information that discusses their concerns, and acquiring community buy-in are all vital for the acceptance and successful implementation of mosquito control and other public health measures in Puerto Rico.” (Rosado-Santiago & Pérez-Guerra [Unpublished]). There is limited literature about the planning and implementation of community-based projects that assess performance of *Ae*. *aegypti* novel vector control methods and measure incidence of disease transmission. This article includes formative research conducted before the implementation of the new project called Communities Organized to Prevent Arboviruses (COPA) in Ponce, Puerto Rico, demonstrating important community perceptions and strategies to consider for effective project implementation and helping to close the gap of limited literature on this topic, including literature focused on Puerto Rico.

The COPA project aims to work with communities in Ponce, Puerto Rico, to assess the effectiveness of vector control interventions, specifically the impact of *Wolbachia* suppression in reducing mosquito populations and human arboviral infections [42]. The intervention involves the release of male mosquitoes with *Wolbachia* bacterium to mate with wild female mosquitoes, and reduce their population as their mating is incompatible. The COPA project includes a longitudinal cohort [43] of about 3,800 participants established in 2018, who are monitored to determine arboviral diseases incidence through annual serosurveys and active weekly acute febrile illness surveillance. Vector control attitudes and prevention practices are assessed through annual questionnaires, and mosquito population densities are estimated through systematic entomological surveillance. As limited information exists on dengue incidence in Puerto Rico, and risk factors at the individual and community level, cohort studies are extremely important tools to determine links between risk factors and incidence of the disease. Assessing community perceptions before the implementation of a cohort could: increase participation and retention rates, increase acceptability of the study in the communities involved, and allow the use of the cohort to include additional relevant health issues if resources allow it [44].

This study aimed to identify potential challenges and solutions for the implementation of the COPA community cohort. Specific objectives were to:

1. Explore the perceptions from residents living on project’s selected communities regarding community geographic limits.
2. Identify health and social issues deemed most critical by participants.
3. Describe communication and decision-making processes of residents in the selected communities.
4. Identify community leaders, volunteers and neighborhood associations and their potential involvement in the project.
5. Examine the importance of mosquito-borne diseases and vector control strategies for participants.

## Methodology

To prepare to carry out the COPA project—designed both as a cohort and a cluster randomized trial with two arms (control and intervention) [42–43, 45]—we used ethnological and qualitative methods to identify communities that would want to participate in the project. COPA is a collaborative project between CDC, Ponce Health Sciences University-Ponce Research Institute, and the Puerto Rico Vector Control Unit (PRVCU).

A behavioral scientist, public health advisor, project coordinator, two health educators, and two case managers participated in assessing community needs and perceptions. In addition, we conducted reconnaissance visits using participatory and non-participatory observation techniques [46], informal interviews (II), focus group discussions, (FGD) and in-depth interviews (IDI) [47–49].

### Location selection

In summer 2017, we made reconnaissance visits to the six communities in Ponce (S1 Table) that were initially proposed by COPA researchers, based on arboviral diseases incidence obtained through the Sentinel Enhanced Dengue Surveillance System (SEDSS) data [50–52]. Ponce is a municipality in southern Puerto Rico with an estimated population of 113,401 in 2017 and 31 neighborhoods (*barrios*). During the visits, we obtained information to describe types of neighborhoods and housing (e.g., urbanizations, land plots, walkups, gated communities; public housing; wood, cement); places most frequently visited by residents within the communities, community facilities (e.g., parks, community centers, sports and health facilities, schools, churches). We equally conducted three informal interviews in one community with the following community members: a participant identified from previous studies, a small business owner, and a community leader. The leader provided us with information on the best strategies for contacting residents and names of other community leaders with whom we could collaborate with and who could serve as liaisons to the residents. Shortly after these visits, Hurricane Irma and Maria made landfall in Puerto Rico in September 2017. As a result, visits to the communities were halted until October 2017 when visits were resumed to recruit potential participants for interviews.

### Selection of participants

We conducted FGDs with community leaders and residents of the six proposed communities. Researchers identified participants of a recent Zika research project conducted by PHSU-PRI and CDC [52]; using the snowball technique [53], these participants in turn, identified their community leaders. We then made phone calls or visited potential participants to invite them to the FGD sessions and provided them with information on COPA objectives.

Similarly, we interviewed community leaders identified using snowball sampling. This is that community leaders who participated in the previous FGD identified other community leaders to participate in the IDI.

### Focus group discussions and in-depth interviews

FGD and IDI sessions were conducted in Spanish because it is the language mostly spoken in Puerto Rico. Before starting the FGD or IDI sessions, participants provided verbal consent to: participate in the session or interview, and for researchers to record audio, and take notes of the discussion.

The moderator used a guide of 20 open questions to lead the FGD. The FGD guide was similar to the IDI guide because we wanted to consistently understand the various perspectives of residents and community leaders on the questions asked. The questions were based on the study objectives including: communities’ geographic and social composition, communication channels, leadership and organizations, communities’ important problems; and individual, government and private sector awareness; and mosquito-borne diseases prevention. (See S1, S2 Files). We conducted FGD and IDI until saturation of participants’ comments was achieved; that is when participants’ answers are repeated and the capacity to get new information has been reached [47, 49].

### Content analysis of FGD and IDI

We used audio files to fill the notes with the most relevant comments and complete verbatim transcriptions. For the analysis, four research staff coded the notes and transcripts individually using the MAXQDA 12 program; and the participants’ comments were the unit of analysis. To do so, researchers performed “open coding” to establish the codes and “axial coding” to relate the codes to each other [49] and met to agree upon codes to include in the final analysis. We developed the categories of analysis based on the research objectives and participants’ comments. We then merged related categories to develop the final discussion topics. Finally, we used the grounded theory method to perform a systematic inductive analysis of the data, identifying patterns that explain the research results [49].

### Ethics statement

This study was reviewed and approved by the Ponce Medical School Foundation, Inc. Institutional Review Board, approval number 171110-VR. Participants provided verbal consent before focus group discussions and interviews.

## Results

We conducted 14 FGD in two rounds due to the expansion of the COPA project to additional geographic areas in Ponce after the first round was completed. The first round was held from October to December 2017 and the second from April to May 2018 with a total of 80 residents and community leaders. We conducted 16 IDI with leaders who had not participated in the FGD from six communities. The moderators and notetakers analyzed 28 sets of notes from the FGD and 15 IDI; one IDI was excluded because of invalid responses. We developed 12 categories for the analysis of FGD and IDI using the research objectives and participant feedback. We summarized the results in eight themes below, which reflect the challenges and potential solutions participants perceived for implementing COPA project activities.

### Theme 1: Community geographic delimitation

Both residents and leaders of the FGDs and the IDIs indicated that the six communities mentioned initially by the interviewers were *barrios* (S1 Table) each consisting of up to 12 communities. For this reason, we termed the larger areas as *barrios*. Some *barrios* were so large that they could be considered a town on their own. They had commercial premises, government agencies, colleges, hospital facilities; one *barrio* covered an entire ZIP code. The leaders in the IDIs delved into the number of dwellings and types of communities within their *barrios* (gated and open urbanizations, plots of land, and public housing projects) and described their facilities and recreational areas. Below we included some quotes from the participants’ interviews:

* Final clusters were established from the six initial *barrios* and other Ponce areas to reach the 38 clusters required for the study sample.

- *“#1 is a community in progress*, *a community that has the characteristics of a village*. *By this I mean that it has its own [ZIP] code…*, *it has a hospital*, *university*, *businesses…”*
- *“The neighbors have known each other for years… they are people who live here from generation to generation*, *except [for those who live in] housing complexes that were built [later]*, *the housing estates and the buildings and apartments*. *…most of the residents live on plots of land [parcelas]… there are many [houses in the barrio]*, *exceeding 1*,*000 [houses]…”*

### Theme 2: Social description of communities

FGD and IDI participants mentioned that the *barrios* were *“quiet because there are many elderly residents*.*”* Some communities housed several generations of families, i.e., grandparents, parents, and grandchildren. In such communities there were more young people between 13 and 18 years old.

- *"Our demographics are mostly older citizens because this*… *community was founded almost 40 years ago*… [in other communities] *we have few young people in the 13 to 18 age range*…*"*

### Theme 3: Neighborhood organizations and influential people in communities

FGD participants and IDI leaders explained that not all *barrios* or communities have community organizations or experienced community leaders. In these settings leaders identified people in charge of sports facilities or community centers as those best suited to solve some problems. After Hurricane Maria, some of the community organizations and associations recognized by community members (S2 Table) no longer existed, and many of the people who had leadership roles within the *barrios* were no longer in those roles because older leaders retired, and others moved out of the communities. In communities with no leader, neighbors carried out street meetings to discuss the best course of action to solve problems and decide who would be responsible for its implementation. Some participants informed

- *“*…*the couples here [participating in the focus group]*, *all our children have left…to the United States*, *my sisters*, *my brothers*, *and so on*,… *so*, *we are by ourselves*. *You know*, *each one with their ailments and illnesses*.*”*
- *My brothers started playing baseball here… at that time there was a lot of youth and children who liked baseball… My brother created a large group [baseball team]*, *but there was no movement to select a president for baseball activities… because … as they reach their twenties and get married*, *they make their own lives*. *He [her brother] married*, *made his life and my other [brother] is also making his life in the United States*.*"*

People recognized as having influence in community dynamics and organizations included unaffiliated community leaders, the leadership of community organizations that remained functional after the hurricane, the administrators of communities’ Facebook pages, small business owners, and gatekeepers like community volunteers, religious leaders, and teachers.

### Theme 4: Most important communication channels in the communities

Before Hurricane Maria, leaders and residents held meetings convened through: loudspeakers, house-to-house visits, and word of mouth. Community organizations distributed flyers at bakeries and churches and placed posters and banners in communal locations. Communities also used Ponce regional media like *Radio Católica* and *Periódico La Opinión* to promote community activities, expose community problems and to seek government help in finding solutions. After the hurricane, communities increased the use of email, texting, and social media networks such as Facebook (e.g., *Noticias de Ponce*, *a digital newspaper*), WhatsApp and Instagram. As cited by participants, these are the best ways COPA can use to communicate with community members of all ages.

- *“With a loudspeaker…”*
- *“And [through] the Internet…”*
- *“… when there is a broken pipe in the road … I communicate with Noticias de Ponce; I send photos of the road like this…”*
- *“They put flyers in the [community] center and we continue spreading the word; that there will be an activity in that place*.*”*

### Theme 5: Important problems that affected the communities

According to leaders participating in IDI, the most important community problems were infrastructure conditions, health issues, crime and drugs, poverty, and elder assistance needs. Infrastructure and health problems worsened after Hurricane Maria due to lack of help from the municipal and central governments; FGD participants mentioned accumulation of debris, and insufficient insecticide spraying, as well as problems with standing water and mosquitoes. Participants said that due to the lack of spraying, residents used alternative measures such as creating smoke with bonfires to eliminate mosquitoes. In turn, participants associated campfires with worsening asthma and sinus conditions among residents. Accumulated trash attracted rats, vectors of leptospirosis, which was another concern for the participants. Problems with clogged sewers, flooding, and broken pipes, clandestine landfills, and inadequate tree pruning, and weeding worsened after the hurricane.

- *“We are talking with the municipality to see if they help us with that … They [residents] throw garbage here into the water canal… that*, *would affect mosquitoes and other insect breeding sites… I would tell you that this is a mosquito warehouse*.*"*

Other important community problems were related to the lack of electrical power in houses and for streetlights, bedridden elderly people, and mental health problems. The lack of electricity and police patrols aggravated the problem of drug use, crime, and vandalism, as well as the delay in the provision of services by government agencies (see Table 1). Crime, in addition to the lack of electricity and police patrols generated lack of trust from residents careful of strangers in their communities, as illustrated below:

- *"*… *there are many people alone*, *elder people*.… *we are very alert so that nothing happens to those people*. *We cannot let anyone in*. *Anyone who enters an urbanization has to provide an identification*, *so I can be sure that there is no type of danger*,… *"*

**Table 1:** Important problems in the communities mentioned by participants of the focus groups discussions and the in-depth interviews.

**Types of problems**	**Items associated with type of problems**
Infrastructure	Deteriorated roads and structures
	Floods
	Interruption of water and electric services
	Lack of illumination and streetlights
	Lack of pipe water pressure
	Sewage/broken pipes
	Leaks in the roof of community centers
	Lack of recreation/maintenance areas
Social	Crime /drugs/unsafe streets school dropouts
	Job shortage/poverty
	Lack of leaders
	Accumulation of debris and trash
	Elderly assistance needs/bedridden elderly
	**After Hurricane**
	Loss of homes/abandoned houses eviction
	Food scarcity
Health	Mosquitoes
	Street animals (horses, dogs, pigeon droppings)
	Asthma from pollution
	Diabetes
	Cardiovascular diseases
	Mental health
	**After Hurricane**
	Leptospirosis due to debris and rats
	Food poisoning from unrefrigerated food

### Theme 6: Residents’ awareness about mosquito-borne diseases

FGD and IDI residents and leaders acquired information on arboviral diseases from family members, neighbors, and health providers (e.g., home nurses, hospital staff, lactation educators) who distributed handouts and flyers. They also received information from traditional media such as television and radio, newspapers, and municipal officials. Regarding mosquitoes, the most relevant topics to the COPA project included curiosity about where mosquitoes come from and their most common breeding sites in the *barrios*, the increase in mosquito numbers, how to control mosquitoes, and how to prevent bites. Participants also mentioned the severity and duration of symptoms associated with Zika, dengue, and chikungunya when a neighbor or relative fell ill and the incidence of these diseases in their communities.

- *“At the health fair… I got Zika information that the municipality distributed*.*”*
- *"These days*, *what we hear the most about is dengue*.*"*
- *"[Health inspectors] … came … to check how we keep the containers*.*"*

### Theme 7: Actions by residents to prevent mosquito-borne diseases

Participants of the FGDs and IDIs said that to prevent mosquito bites they frequently spray insecticides and use repellents (including homemade ones), bonfires, Citronella, and insecticide coils. Some leaders added that to avoid mosquitoes they used screens, fans, bed nets, avoided being outside their homes, and wore clothing that covered their arms and legs. To avoid mosquito reproduction, they eliminated containers with water, cut the grass, and kept their properties and surroundings clean. Some residents set mosquito traps, including homemade traps. Some stayed informed with educational materials and attended health talks.

Other participants claimed that neighbors did not act individually or collectively to prevent mosquito bites and breeding sites. This resulted in people’s disbelief that mosquito breeding could be prevented.

- *"I don’t think [mosquitoes can be avoided]*, *because we live on a tropical island*, *and there will always be mosquitoes*.*"*

Some leaders suggested creating committees to work on mosquito problems. These committees could include employees of the municipality and other entities. The committees could establish community groups to help older adults and people with disabilities dispose of water containers.

### Theme 8: Role of government and private institutions in the prevention of mosquito-borne diseases

When asked about the role of the government and private sector, participants thought the government was not providing the necessary services, was inactive, and that municipal, central, and federal governments were not well integrated, resulting in lack of action on mosquito control. Residents and leaders suggested the role of both municipal and central government, which should be to: fumigate the streets, clean the surroundings of debris and tires, and educate the public on the prevention of mosquito-borne diseases. IDI leaders added that municipalities should inspect yards.

- *“… [the municipality should] put staff cleaning all the streets*. *They had it before*, *but they suspended it*.*"*
- *“Spraying*, *the main thing is to spray*. *They [the municipality] used to come and spray*.*"*
- *“It would be great to send staff to this neighborhood*, *from you [the municipality]*, *to check the environment*, *not inside the houses*, *but around*, *and right there give an orientation to residents as to why they are doing this*… *because we are really worried*!*"*

As a solution, IDI participants said the central and federal governments could oversee mosquito control activities and allocate adequate funds and human resources for municipalities to implement mosquito control programs and promote education and prevention. The leaders suggested that the federal government should also offer orientation and technical assistance to the central and municipal government, create proposals to bring funds, bring specialists and carry out studies on arboviral diseases and mosquito control. In agreement with FGD participants, leaders understood that the role of nonprofit organizations is to work with the government and the private sector to educate the public.

- "*That [the federal government] is the one that has to supervise*.*"*
- *"Well*, *Uncle Sam*, *we need the money*. *These are the funds to carry out this [type of project] …*
- *“The federal government has a Health Department*, *just like the central one*, *just like the municipal one*. *Those three Health Departments must work together to help… Puerto Rico*. *Because there are three agencies*, *they can help significantly*, *if they communicate with each other*. *Yes*, *it is a lack of communication that affects things*.*"*

### Discussion

This study found that communities’ organization in the intended areas of the COPA project was limited, due to recent natural disasters along with political and economic conditions that have led to an increase in population migration to other areas of Puerto Rico and the continental United States for better living conditions. This helped us identify emerging community leaders willing to collaborate with COPA. Migration also influenced the age composition of communities toward an older population. Therefore, researchers modified age criteria and used communication and promotional strategies to better recruit and retain participants.

Knowing leaders’ and residents’ opinions, perceived challenges, and their proposed solutions was important to design and implement the community cohort study to assess arboviral disease incidence. It was also necessary to assess the effectiveness of vector control methods like mosquitoes with *Wolbachia* suppression. This study helped determine the geographic delimitation of the intended participant communities, which, along with other information on disease incidence and spatial distribution, informed cluster delimitation for the project.

For an arboviral cohort study that includes dengue incidence, age distribution is important to determine disease incidence more precisely, as by a certain age, most residents of endemic areas have been infected with dengue at least once. We were expecting to find diverse age groups among the *barrios’* population to recruit children for a dengue cohort study, but we found that a significant number of community residents were older adults. An analysis of the 2010 dengue epidemic in Puerto Rico showed that the most infected age group was children from 10 to 19 years old, suggesting that many people in Puerto Rico have had a dengue infection before adulthood [54, 55]. Our data helped understand the challenges of establishing a cohort with younger participants in Ponce. Along with previous epidemiological surveillance data, researchers informed the determination of the sample inclusion criteria for the serological survey. Included were residents aged 1 to 50, as opposed to only children 1 to 19 years of age.

Our study identified that population attrition in the communities after Hurricane Maria posed challenges for cohort study recruitment. A study in Puerto Rico found that migration after the hurricane was temporary, while migration due to socioeconomic struggle was permanent [56]. A decreasing population and communities composed mainly of older adults underscored the importance of devising participant recruitment and retention strategies that were later used in the COPA study. The strategies were monetary incentives, a promotional campaign in traditional and social media, and more recently, drive-through testing during the 2019 coronavirus disease (COVID-19) pandemic. Similarly, other studies in Bangladesh, China, Peru, and Thailand have offered tests [57], small incentives such as mosquito repellent [58], and transportation [59] to improve participation.

Through our formative research activities, we found that residents’ migration resulting in more abandoned houses [60]—which in turn increases the number of mosquito breeding sites on these properties—was a concern for community residents, and creates a challenge for effective mosquito surveillance and control in this area. These findings, along with results of entomological surveillance conducted in the area confirmed a high mosquito population in Ponce, stressing the need to test and use new mosquito control methods for *Ae*. *aegypti* control.

Crime and drugs in certain parts of the communities were important challenges mentioned that could risk the safe and consistent implementation of serological and entomological surveys in the studied communities. Movement within the communities could be restricted due to crime, and residents are suspicious of the presence of strangers. A study using insecticidal net screens in Mexico encountered lack of trust from residents to allow project staff into their homes due to strangers impersonating health staff [35]. Violence due to the use of narcotics was also reported in the Camino Verde project in Mexico, and residents were recruited as brigade members to make house visits [29]. In response to this concern, COPA recruited community leaders as promoters to assist the work in the communities. To increase trust, the COPA community coordinator along with community promoters and leaders have established and maintained communication channels (phone calls, text messages and chat groups) to raise field staff awareness of safety issues related to crime that could affect project data collection in communities affected by violence. When misunderstandings occur in the field, community leaders and promoters also serve as mediators with community residents.

As expected, Hurricane Maria changed the dynamics in these communities, and our activities allowed us to see more clearly how these changes affected local leaders and the project implementation. Even before Hurricane Maria, only some communities had a neighborhood association, and many leaders were older. After the hurricane, other leaders had moved out of the communities or migrated to the U.S. mainland due to the hurricane’s destruction. The absence of leaders could have been a challenge to COPA because the project needed to consult decisions with the leaders and promote the project within the communities. As a solution, other residents assumed leadership roles. We identified these new leaders and created a list of residents who took on leadership roles such as custodians of community facilities, developers of community Facebook pages, participants in neighborhood security groups, and people trusted by residents. The leaders became COPA spokespersons explaining the purpose of the project and its benefits. They reviewed activity plans and educational materials, gave their opinions about innovative activities to control *Ae*. *aegypti* [21–27, 61–65], coordinated meeting site, summoned residents for meetings, and improved residents’ trust to increase participation in serological and entomological surveys. Some of those leaders were employed as community promoters to carry out the project orientation and education activities. The Camino Verde study in Mexico used a similar approach to increase participation and trust recruiting *brigadistas* to work with community member groups, educate the community on *Ae*. *aegypti* biology and control, and conduct household surveys and yard inspections [29].

Another important outcome of this formative research was participants’ perception that neither the residents nor the governments (municipal, central, or federal) took proactive or coordinated actions to improve the mosquito problem and prevent arboviral diseases. Similar concerns have been reported by studies in Vietnam [34] and Ecuador [38] in which participants perceived lack of coordination, communication, policy guidance, and engagement of government institutions and lack of social involvement in dengue control programs. Participants suggested creating community committees and integrating government officials and other entities. Following this recommendation, we identified scientists from PHSU and PRVCU, municipal and central government officials from the different departments (Puerto Rico Department of Health, Department of Natural Resources, Environmental Quality Board, etc.). We also identified community leaders interested in collaborating with the project to form community advisory committees and a central advisory committee who would offer their expertise to carry out studies related to the reduction of *Ae*. *aegypti* populations. The committees focused on the need to reduce the risk of contracting arboviral diseases, a recurring public health problem in the communities given the ongoing conditions favorable to *Ae*. *aegypti* abundance [66, 67]. Particularly, the community advisory committees reviewed and tested educational materials while the PRVCU Leadership Board and Technical Committee discussed and provided suggestions on the vector control strategies to be tried out.

With the participants’ suggestions about the best action to share information and promote project activities, we developed a community engagement plan with a communications plan. The communications strategy to promote COPA included traditional and social media channels to inform the older and younger community residents. Traditional methods to spread information like house visits were preferred because they provided face-to-face and individual interaction to answer questions. Loudspeakers seemed to be the best strategy to promote project and recruitment activities in each cluster. Similar results were found in Cambodia whose preferred methods by community residents were 1) house visits during which residents could clarify their doubts and 2) the use of loudspeakers to share information and promote activities [31]. *Noticias de Ponce*, a digital newspaper in Facebook, and other Ponce media, promoted the cluster house visits to recruit participants for the COPA cohort. In addition to the community engagement plan, the results presented in this article were used to inform and improve the development of knowledge and practices sections of the annual COPA questionnaire. After using the communications strategy, no opposition to the project activities has taken place [42].

Our results have some limitations. These results are particular to the participants in Ponce, Puerto Rico and cannot be generalized. Human perceptions may differ from one place to another, but there are still certain areas of similarities. Lessons learnt could be adopted in projects that share similarities.

## Conclusion

This study explored how natural disasters and migration may impact a community-based project to evaluate a novel method for controlling *Ae*. *aegypti*. We found that Hurricane Maria, along with prior migration, could have influenced the movement of the younger population out of the communities while most older adults remained. The hurricane also hampered the establishment of community groups and leadership. This study also informed the COPA project by refining geographic areas based on community barrios, identifying potential implementation challenges in advance, and informing the development of a comprehensive communications and community outreach plan. This study provided a solid foundation for the COPA project based on building trust with communities.

## Supporting information

S1 File Guide of questions for moderator to conduct focus group discussions to assess the acceptability and feasibility of *Aedes aegypti* control strategies with residents from Ponce, Puerto Rico. (PDF)

S2 File Guide of questions for moderator to conduct interviews to assess the acceptability and feasibility of *Aedes aegypti* control strategies with community leaders from Ponce, Puerto Rico. (PDF)

S1 Table List of the initial six barrios proposed, and *38 final community clusters selected, for the COPA project in Ponce, Puerto Rico. (PDF)

S2 Table Community Organizations of COPA *Barrios* mentioned in focus group discussions and in-depth interviews. (PDF)
