# Supplementary material for: Community perceptions on challenges and solutions to implement an Aedes aegypti control project in Ponce, Puerto Rico (USA)
Source: PLoS One. 2023 Apr 17;18(4):e0284430. doi: 10.1371/journal.pone.0284430 (PMC10109480; doi:10.1371/journal.pone.0284430)
Supplement: S2 File — (PDF) [file pone.0284430.s002.pdf]

Part 1: Community Dynamics

1. How do you describe this community? Up to where does it go?
2. What distinguishes this community and why?
3. Who are the most influential people in this community?
4. Which are the most important problems in this community?
5. How do neighbors come to an agreement and decide that a situation is an important problem in this community?
6. How has the community tried to solve these problems?
7. Which are the organizations in this community?
    a. How have the organizations in this community tried to solve these problems? (If they're unsure what is a community organization, then suggest schools, churches, local businesses that form the function of community spaces, etc.)
8. How do people in this community organize to solve problems?
9. What types of activities are organized in this community?
    a. Out of these, which are the most successful in terms of attendance and participation?
10. How are activities or meetings organized and communicated to the community?
    a. If participants mention word of mouth, does anyone assist in keeping residents informed about activities and meetings that are going to be conducted?
11. Who is the person or people who work most for the health of the people of this community?

Part 2: Vector Control Practices and Interest in Intervention

1. Do residents speak about dengue, chikungunya, and Zika in this community?
    a. When are mosquito-borne illnesses mentioned in the community?
    b. Who have you heard speaking about dengue, chikungunya, and Zika?
2. Are there a lot of mosquitoes in this neighborhood? In what months are there more mosquitoes?
3. Can you tell me, what do residents of this community say about dengue, chikungunya, and Zika?

4. Have you heard about how serious or important people consider diseases transmitted by mosquitoes to be in this community?

5. What do you think individuals can do to prevent dengue, chikungunya, and Zika?
    a. Do you think that preventing Zika, dengue, and chikungunya is something that individual people can do, realistically? Why or why not?

6. What do you or other members of the community already do to protect yourselves from mosquitoes?
    a. Do you think there is more that members of the community could do to protect themselves from mosquitos? Why?

7. What should be the role of municipal, central or federal government in the prevention of dengue, chikungunya, and Zika?
    a. What do you think about the involvement of non-governmental organizations or associations in the prevention of mosquito-borne illnesses?

8. What are your worries or doubts about the strategies that individuals or the government use to control mosquitos?

9. What strategy do you think would work best to control mosquitos in this community?

10. If we were to work in your community, what would motivate you to let a federal health worker or Ponce School of Medicine come into your home?

11. If we need your collaboration, in what types of tasks can you collaborate? and have availability? What hours would you have available?
