# Supplementary material for: Community perceptions on challenges and solutions to implement an Aedes aegypti control project in Ponce, Puerto Rico (USA)
Source: PLoS One. 2023 Apr 17;18(4):e0284430. doi: 10.1371/journal.pone.0284430 (PMC10109480; doi:10.1371/journal.pone.0284430)
Supplement: S1 Table — (PDF) [file pone.0284430.s003.pdf]

*List of the initial six barrios proposed, and *38 final community clusters selected, for the COPA project in Ponce, Puerto Rico*					
**Initial community codes**	**Area in acres**	**Structures**	**Final cluster codes**	**Area in acres**	**Structures**
TU	546	3216	TU01	135	791
			TU02	166	927
			TU03	115	617
PT	292	1764	PT01	101	624
			PT02	98	466
JD	193	1325	JD01	154	1214
PL	372	2187	PL01	112	543
			PL02	134	642
			PL03	73	528
VC	279	2683	VC01	143	1248
			VC02	147	821
CL	482	2251			
New communities			AC01	87	467
			CB01	108	639
			CB02	121	1180
			CT01	158	1064
			CT02	37	253
			CT03	74	491
			DP01	75	586
			GL01	98	600
			GL02	117	651
			GL03	100	709
			JC01	59	459
			JC02	64	462
			JC03	134	943
			MA01	129	517
			MA02	105	598
			MA03	172	1125
			PO01	92	792
			PO02	128	1123
			QS01	243	2187
			RP01	71	151
			SA01	114	598
			SA02	146	975
			SI01	55	280
			ST01	97	824

			ST02	69	555
			VG01	78	675
			VG02	169	1005

*Final clusters were established from the six initial *barrios* and other Ponce areas to reach the 38 clusters required for the study sample.
