# Supplementary material for: Community perceptions on challenges and solutions to implement an Aedes aegypti control project in Ponce, Puerto Rico (USA)
Source: PLoS One. 2023 Apr 17;18(4):e0284430. doi: 10.1371/journal.pone.0284430 (PMC10109480; doi:10.1371/journal.pone.0284430)
Supplement: S2 Table — (PDF) [file pone.0284430.s004.pdf]

Community Organizations of the COPA Neighborhoods		
**Type of Organization**	***Barrio***	**Organization Name**
**Sports**	CT, PT, JD, ST	Sports Leagues, Domino Teams
**Recreational/Cultural**	TU, CT, PL, JD	Musical Band, Recreational Associations,<br>Social Club, Canine Club
**Religious**	CT, PL, JD	Catholic and Protestant Churches
**Community**	TU, PL, JD, PT, ST	Neighborhood Safety Council, Community Boards, Veterans Association,<br>Ornate/Gardening Committees
**Educational**	TU	Sister Isolina Ferré Centers
**Charitable/Service**	PL	Lions Club
**Environmental**	PL	Environmental groups
**Government**	TU	Center for the Aged, Police League
